# Portal vein thrombosis in COVID-19 infection

**DOI:** 10.1186/s40792-021-01173-z

**Published:** 2021-04-08

**Authors:** Stefanie Sinz, Florian Glaser-Gallion, Thomas Steffen

**Affiliations:** 1 Department of General, Visceral, Endocrine and Transplantation Surgery, Kantonsspital St. Gallen, 9007 Saint Gallen, Switzerland; 2 Department of Radiology, Kantonsspital St. Gallen, 9007 Saint Gallen, Switzerland

**Keywords:** Portal vein thrombosis, COVID-19, SARS-CoV-2, Corona, Thrombotic angiopathy, Case report

## Abstract

**Background:**

The COVID-19 pandemic has rapidly spread worldwide. As it is a novel disease, we have less experience in its possible appearances. Predominantly affecting the respiratory tract, about 20–43% patients also present with extrapulmonary manifestations such as coagulation disorders with thrombotic angiopathy.

**Case presentation:**

In our institution, a patient presented to the emergency department with acute abdominal pain which was caused by portal vein thrombosis. As a COVID-19 nasopharyngeal antigen swab few days earlier was negative, we performed several tests to find out its etiology. After all tests were inconclusive and the patient suffered flu-like symptoms 2 weeks before, we repeated COVID-19 molecular testing and received a positive test result. The patient was treated symptomatically and received therapeutic anticoagulation.

**Conclusion:**

A COVID-19 infection can also be present without typical pulmonary symptoms. In patients with severe abdominal pain and new diagnosed portal vein thrombosis, it is important to think of a COVID-19 infection. Also, the reliability of antigen nasopharyngeal swab should be considered critically, especially if performed wrongly. We recommended to perform molecular tests when in doubt. After the diagnosis of portal vein thrombosis, immediate anticoagulation is recommended to reduce the risk of further complications like intestinal infarction.

## Background

The COVID-19 pandemic has rapidly spread worldwide. Predominantly affecting the respiratory tract, about 20–43% patients also present with extrapulmonary manifestations such as coagulation disorders with thrombotic angiopathy [1]. We report a case of diffuse acute abdominal pain caused by portal vein thrombosis due to COVID-19 disease.

## Case presentation

A 38-year-old male patient was admitted to the emergency department with acute and increasing severe abdominal pain in the epigastrium accompanied by nausea and diarrhea. The patient reported a feeling of abdominal pressure for the past 2 weeks. The week before he suffered from recurring fever attacks up to 38.5° Celsius, coughing and painful pleural irritation. A nasopharyngeal swab specimen for SARS-CoV-2 antigen PCR performed by the family doctor the day before admission to hospital was negative. The patient’s history concerning relevant contacts to positively tested COVID-19 patients was negative. The patient’s medical history was unremarkable; especially no past thromboembolic events were reported.

On admission the blood pressure level was 138/89 mmHg, the heart rate was at 93 beats per minute. The body temperature was 36.7 °C and the oxygen level in a pulse oximeter was 98% on indoor air. The examination showed signs for peritonitis with pain and rebound tenderness in the right lower quadrant.

Blood examination showed elevated C-reactive protein at 122 mg/l (standard value: < 8 mg/l) and elevated leukocyte count at 19.5 G/l (standard value: 4–10 G/l). The liver function parameters were normal, as well as the thrombocyte count (Table 1).

**Table 1:** Blood findings on admission

Laboratory findings (standard value)	
Total bilirubin (< 20 μmol/l)	18 μmol/l
Alkaline phosphatase (30–120 U/l)	77 U/l
GGT (< 65 U/l)	66 U/l
LDH (< 265 U/l)	299 U/l
CRP (< 8 mg/l)	122 mg/l
D-dimer (< 0.5 mg/l)	6.87 mg/l
Prothrombin time (0.81–1.43)	0.67
Prothrombinase-induced clotting time (s)	56 s
Hemoglobin (140–180 g/l)	172 g/l
White blood count (4–10 G/l)	19.5 G/l
Platelets (150–300 G/l)	281 G/l

**Fig. 1:**
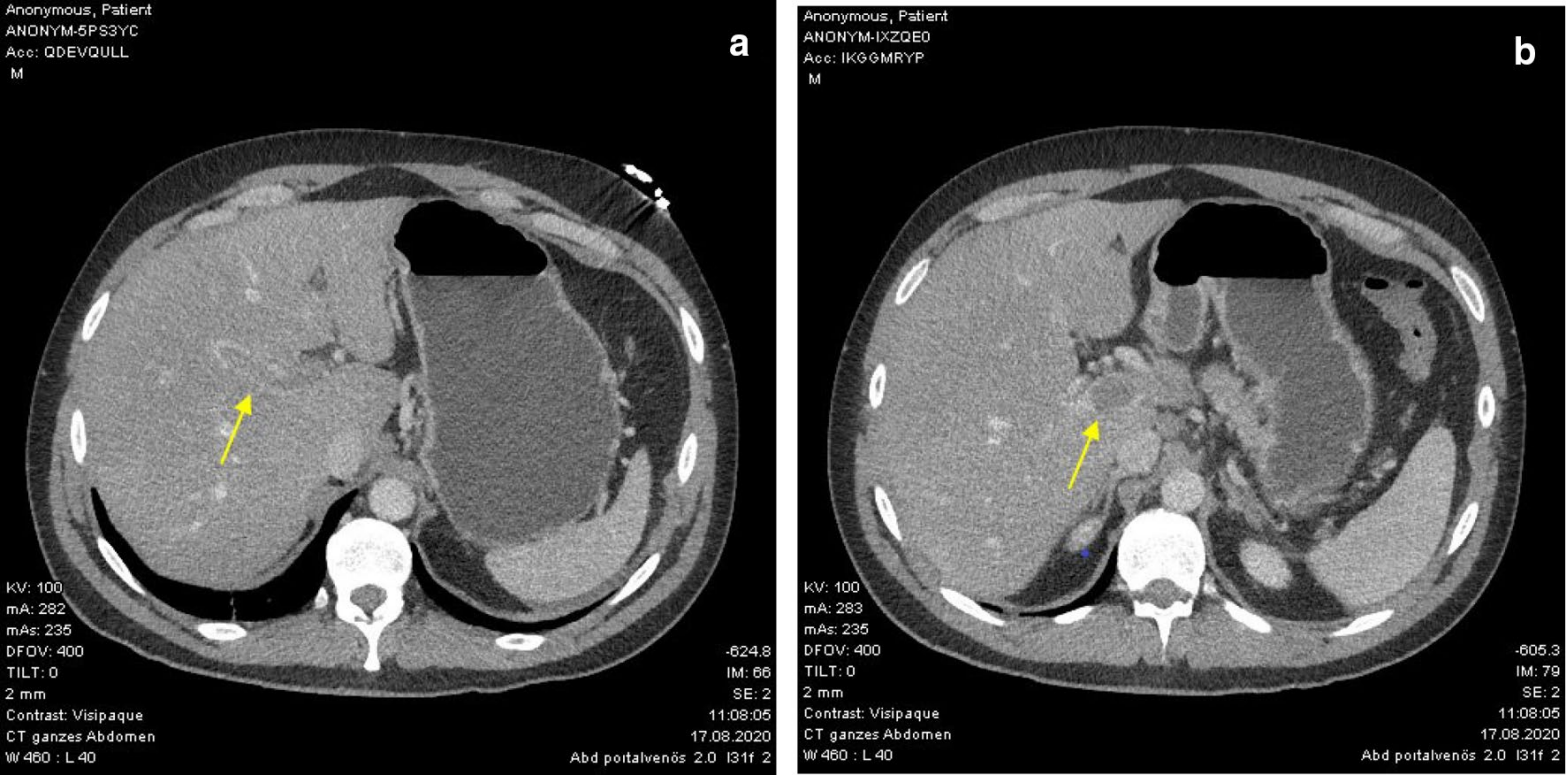
**a**, **b** CT-scan with portal vein thrombosis on admission, which occludes nearly the whole caliber (yellow arrows)

An abdominal ultrasound examination performed in the emergency department revealed dilated intestinal loops. The computed tomography scan (CT) showed an extensive portal vein thrombosis with signs of mesenteric venous stasis and enhanced small bowel segments in the left abdomen (Fig. 1).

The patient was admitted to intensive care unit for observation, and we started continuous intravenous anticoagulation with unfractionated heparin (target PICT 75–110 s). As no apparent reason for portal vein thrombosis was initially known, we performed further hematological examinations. D-dimer was abnormal at 6.87 mg/l (standard value: < 0.5 mg/l). Except a heterozygous prothrombin variant, we found no evident cause of the thrombosis. Inherited and acquired thrombophilia were excluded. The blood examinations after 2 days revealed three-times elevated serum pancreatic amylase as a sign for mild pancreatitis due to the portal vein thrombosis. Hepatic clarification (ultrasound, liver biopsy and blood work) showed normal liver parenchyma as well as negative hepatitis serology.

As no explanation for portal vein thrombosis was found and the patient reported of flu-like symptoms 2 weeks before, we performed a SARS-CoV2-immunoserology, showing IgM to be positive. A repeated test after 2 weeks showed again positivity for IgM and also for IgG, which confirmed COVID-19 infection in this patient.

**Fig. 2:**
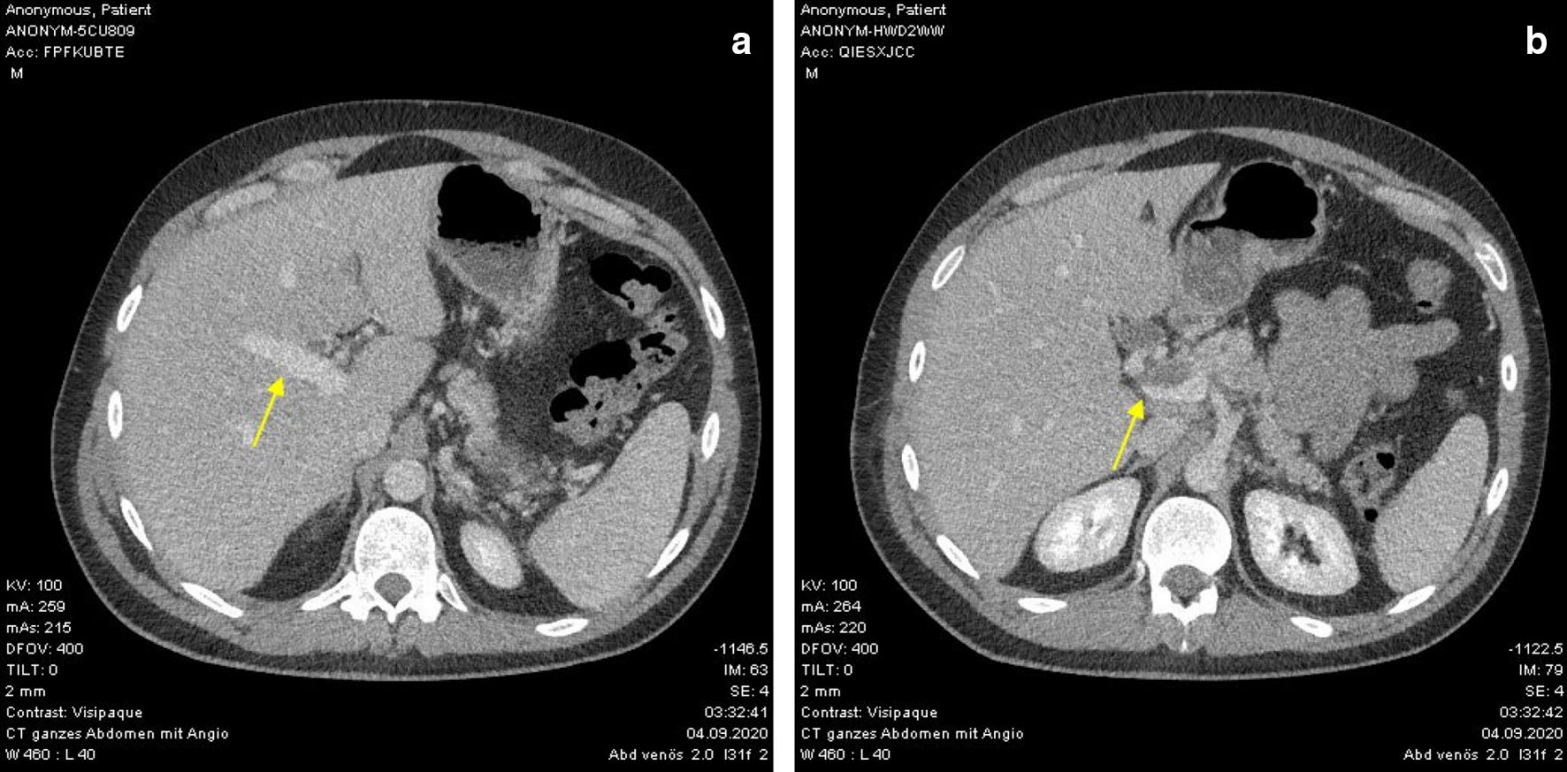
**a**, **b** CT-scan 14 days after admission with regressive portal vein thrombosis (yellow arrows)

Repeated CT 2 weeks later showed regressive portal vein thrombosis under continuous anticoagulation (Fig. 2). On the duplex ultrasound, the portal vein blood flow showed normal scores. The intravenous anticoagulation was changed to Vitamin K antagonist oral anticoagulant (target PT INR 2.5–3.5).

Computed tomography of the chest on admission and after 2 weeks showed normal imaging without any ground-glass opacities, vascular enlargement or bilateral abnormalities.

The patient persistently suffered from abdominal pain, which needed to be treated with a multimodal analgesia regimen.

After improvement of his medical condition, the patient was discharged after 20 days of hospitalization with a Vitamin K antagonist oral anticoagulant for at least 6 months. Further imaging is planned to evaluate the portal vein thrombosis and the duration of oral anticoagulation.

## Conclusion

According to current literature, there are few cases of portal vein thrombosis due to COVID-19 infection [2–4]. In all these case reports, the pulmonary affection is the leading part of the disease; the portal vein thrombosis was described as a comorbidity.

In our case, first the portal vein thrombosis was detected followed by diagnosis of COVID-19 infection. Pulmonary symptoms did not occur in the patient, except the coughing 2 weeks before. Therefore, this is a case with a portal vein thrombosis as the leading symptom for COVID-19 infection.

Patients with severe abdominal pain and known COVID-19 should undergo abdominal imaging. Patients with unknown etiology of abdominal thrombosis and negative antigen swab test should although be excluded COVID-19 infection with immunoserology.

The reliability of the antigen nasopharyngeal swab is uncertain, as inadequate sample taking could have been obtained. Nasopharyngeal swabs need to be deeply inserted to collect an adequate amount of viral RNA. Therefore, these tests should be performed by highly trained staff. Also, false-negative results tend to occur more often with antigen tests than with molecular tests. In our case, an additional COVID-19 test when the patient was admitted to the emergency department would have made sense.

After the diagnosis of portal vein thrombosis, immediate anticoagulation is recommended to reduce the risk of further complications like intestinal infarction. Especially ICU-patients with COVID-19 benefit from early therapeutic anticoagulation [5]. Liver enzymes should be monitored, on the one hand due to portal vein thrombosis per se and on the other hand due to possible direct cell damage caused by viral infection [6].

## Data Availability

Not applicable.
